# Using Large Language Models for Chronic Disease Management Tasks: Scoping Review

**DOI:** 10.2196/66905

**Published:** 2025-09-29

**Authors:** Henry Mukalazi Serugunda, Ouyang Jianquan, Hasifah Kasujja Namatovu, Paul Ssemaluulu, Nasser Kimbugwe, Christopher Garimoi Orach, Peter Waiswa

**Affiliations:** 1 Department of Information Technology, School of Computing and Informatics Technology, Makerere University, Kampala, Uganda; 2 School of Computer Science and School of Cyberspace, Xiangtan University, Engineering Building, 2nd Floor, Yuhu District, Xiangtan, Hunan, 411105, China, 86 73158292718 ext 186; 3 Department of Information Systems, School of Computing and Informatics Technology, Makerere University, Kampala, Uganda; 4 Department of Computer Science, Faculty of Computing and Library Science, Kabale University, Kabale, Uganda; 5 Department of Networks, School of Computing and Informatics Technology, Makerere University, Kampala, Uganda; 6 Department of Community Health and Behavioral Sciences, School of Public Health, College of Health Sciences, Makerere University, Kampala, Uganda; 7 Department of Health Policy Planning and Management, School of Public Health, College of Health Sciences, Makerere University, Kampala, Uganda

**Keywords:** chronic diseases, disease management, artificial intelligence in health care, large language models, natural language processing, NLP, generative pre-trained transformer, GPT

## Abstract

**Background:**

Chronic diseases present significant challenges in health care, requiring effective management to reduce morbidity and mortality. While digital technologies like wearable devices and mobile applications have been widely adopted, large language models (LLMs) such as ChatGPT are emerging as promising technologies with the potential to enhance chronic disease management. However, the scope of their current applications in chronic disease management and associated challenges remains underexplored.

**Objective:**

This scoping review investigates LLM applications in chronic disease management, identifies challenges, and proposes actionable recommendations.

**Methods:**

A systematic search for English-language primary studies on LLM use in chronic disease management was conducted across PubMed, IEEE Xplore, Scopus, and Google Scholar to identify articles published between January 1, 2023, and January 15, 2025. Of the 605 screened records, 29 studies met the inclusion criteria. Data on study objectives, LLMs used, health care settings, study designs, users, disease management tasks, and challenges were extracted and thematically analyzed using the Preferred Reporting Items for Systematic Reviews and Meta-Analyses extension for Scoping Reviews guidelines.

**Results:**

LLMs were primarily used for patient-centered tasks, including patient education and information provision (18/29, 62%) of studies, diagnosis and treatment (6/29, 21%), self-management and disease monitoring (8/29, 28%), and emotional support and therapeutic conversations (4/29, 14%). Practitioner-centered tasks included clinical decision support (8/29, 28%) and medical predictions (6/29, 21%). Challenges identified include inaccurate and inconsistent LLM responses (18/29, 62%), limited datasets (6/29, 21%), computational and technical (6/29, 21%), usability and accessibility (9/29, 31%), LLM evaluation (5/29, 17%), and legal, ethical, privacy, and regulatory (10/29, 35%). While models like ChatGPT, Llama, and Bard demonstrated use in diabetes management and mental health support, performance issues were evident across studies and use cases.

**Conclusions:**

LLMs show promising potential for enhancing chronic disease management across patient and practitioner-centered tasks. However, challenges related to accuracy, data scarcity, usability, and ethical concerns must be addressed to ensure patient safety and equitable use. Future studies should prioritize the integration of LLMs with low-resource platforms, wearable and mobile technologies, developing culturally and age-appropriate interfaces, and establishing robust regulatory and evaluation frameworks to support safe, effective, and inclusive use in health care.

## Introduction

Chronic diseases, such as diabetes, heart disease, asthma, lung disease, depression, hypertension, Alzheimer disorder, and cancer, are a significant global burden on health care systems [1-3]. These conditions often lead to long-term health issues and have profound physical, psychological, and social impacts on patients [1,2]. Chronic diseases demand continuous, personalized care, often resource-intensive and difficult to scale [1]. Therefore, disease management, which encompasses screenings, regular check-ups, monitoring, coordination of treatment, medication adherence, lifestyle modifications, and patient education, is crucial for improving patient outcomes, enhancing quality of life, and reducing the overall burden on health care systems [1]. However, the resource-intensive nature of personalized, continuous care often makes it inaccessible to many patients, particularly in underserved populations where limited access to health care professionals and resources creates significant barriers to effective disease management [3].

In recent years, the use of digital technologies such as wearable devices [4], mobile apps [5], and chatbots [6,7] has grown significantly in the management of chronic diseases. These have mainly been used for health care tasks, including patient education, symptom monitoring, and medication management [4-7]. The recent emergence of large language models (LLMs) such as GPT, Palm, Llama, and LaMDA [8-11] has demonstrated growing potential in health care applications. These models have been applied in health care for tasks such as personalized treatment recommendations, medical diagnosis, medical record summarization, and interpretation of clinical data to support clinical decision-making and disease management [12-16].

Chronic disease management requires continuous monitoring, patient education, treatment coordination, and personalized care strategies [1]. Recent advancements in LLMs have introduced new possibilities for improving these tasks. For instance, ChatGPT has been explored in providing personalized health advice, enhancing patient engagement, and supporting symptom monitoring [12,13]. In diabetes management, GPT-based models have been investigated for interpreting continuous glucose monitoring data, providing personalized lifestyle recommendations [17], and assessing individualized risk profiles for complications such as retinopathy [17]. Beyond diabetes, LLMs such as LLaMA and GPT have been investigated for mental health support [18], blood pressure measurement using wearable bio signals [19], management of sickle cell anemia [20], and dissemination of information on inflammatory bowel diseases to patients and health care professionals [21].

Despite these applications, several challenges affect the effectiveness of LLMs in chronic disease management, including inaccurate responses, limited and biased datasets, and ethical concerns [17,22,23]. These issues raise concerns regarding the accuracy, reliability, and clinical applicability of LLM-generated recommendations [24]. Given these challenges, a comprehensive review is essential to assess the current applications, identify key limitations, and propose strategies to enhance the effectiveness and safety of LLMs in chronic disease care. While existing reviews explore LLMs in general health care, this scoping review uniquely focuses on their role in chronic disease management. It synthesizes evidence across patient- and practitioner-centered applications, domain-specific challenges, and provides actionable recommendations. Specifically, this review aims to evaluate the current applications of LLM in chronic disease management tasks, identify key challenges, and provide actionable recommendations to address identified challenges.

## Methods

### Search Strategy and Information Sources

This scoping review explored the use of LLMs in chronic disease management following the PRISMA-ScR (Preferred Reporting Items for Systematic Reviews and Meta-Analyses Extension for Scoping Reviews) [25]. A comprehensive search was conducted in PubMed, Scopus, IEEE Xplore, and Google Scholar, selected for their coverage of peer-reviewed medical, technical, and AI-related research. Google Scholar was included to capture a broad range of academic publications, including preprints and conference papers that may not be covered by traditional databases. Search terms included combinations of “Large language models,” “LLMs,” “ChatGPT,” “chronic diseases,” and “chronic disease management.” The search targeted both published and unpublished English-language articles from January 1, 2023, to January 15, 2025, ensuring a focus on recent advancements in LLM applications in health care.

### Article Selection

**Figure 1.**
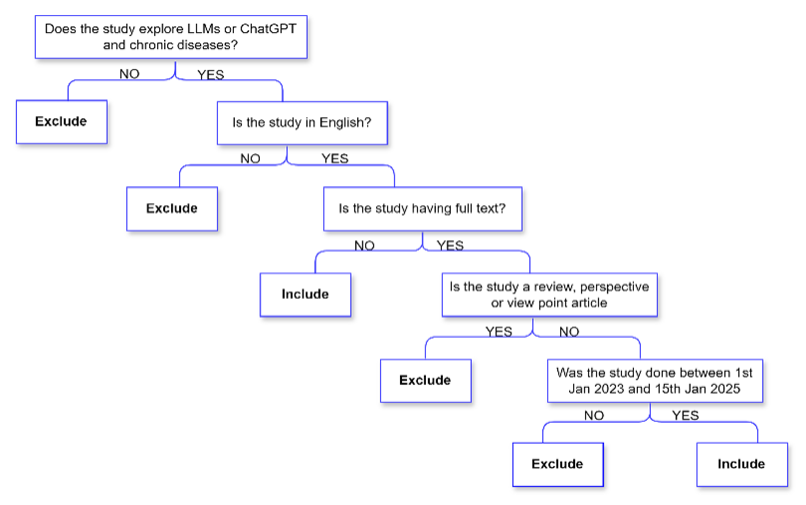
Decision tree for assessing article eligibility. The exclusion of certain publication types was necessary to ensure the review focused on primary research and empirical studies that directly address the application of large language models in chronic disease management.

Studies were included if they focused on applications of LLM in chronic disease management, provided full-text access, were published in English, and appeared between January 2023 and January 15, 2025. Exclusion criteria eliminated nonprimary research (reviews, editorials, viewpoints, and commentaries), abstracts without full text, non-English publications, and articles outside the date range. To capture emerging research and potentially studies, reputable non-peer–reviewed preprints from established repositories such as arXiv and medRxiv were included. Figure 1 illustrates the eligibility screening process with a decision tree.

### Data Extraction and Synthesis

The data from selected studies were extracted into a structured form that captured study objectives, the LLMs used, health care settings, study designs, disease management tasks, identified challenges, evaluation methods, and target users. These extracted data points were selected to ensure a structured and objective analysis aligned with the study’s aims. Study objectives provided insight into the intended applications of LLMs in chronic disease management, while health care settings contextualized their use across clinical and patient-centered environments. Study design and evaluation methods were included to assess methodological rigor and the validity of findings. In addition, data on LLM models, disease management tasks, and key challenges enabled a systematic evaluation of current applications, limitations, and areas for future research.

The Mixed Methods Appraisal Tool (MMAT, version 2018) [26] was used to perform a formal methodological quality assessment. The MMAT was selected due to its flexibility in evaluating diverse study designs included in the review. Two reviewers independently appraised each study using the 5 criteria relevant to its methodological design, with discrepancies resolved through discussion and reached consensus on final ratings. Consistent with scoping review methodology, the studies were not excluded based on quality, but results inform the interpretation of findings [25]. The findings from the included studies were synthesized and presented in alignment with the study objectives. A thematic analysis approach was used to categorize qualitative insights, grouping findings into patient-centered tasks, practitioner-centered tasks, and challenges. Discrepancies in data interpretation were resolved through consensus among the reviewers. Reference management and citation generation were conducted using Mendeley.

## Results

### Included Studies

**Figure 2.**
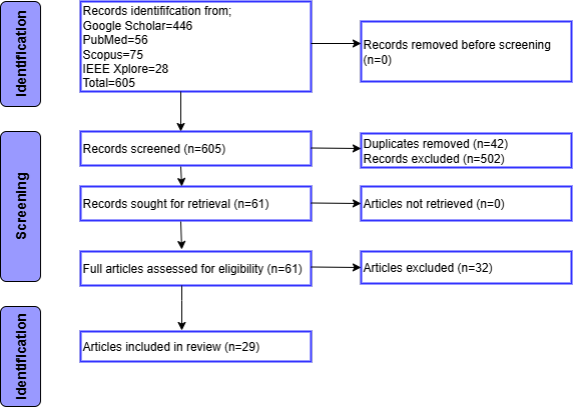
PRISMA (Preferred Reporting Items for Systematic Reviews and Meta-Analyses) flowchart.

The PRISMA flow diagram (Figure 2) outlines each stage of the study selection process. The PRISMA-ScR framework was followed to ensure transparency and reproducibility, incorporating detailed search strategies and clearly defined exclusion criteria. A total of 446 records were identified from Google Scholar, 75 from Scopus, 56 from PubMed, and 28 from IEEE Xplore. After removing duplicates, 242 unique records underwent title and abstract screening, resulting in 61 articles for full-text review. Following the application of eligibility criteria, 29 articles were included in the final analysis.

### Characteristics of Included Articles

Table 1 provides the characteristics of the 29 articles included in this scoping review. The articles were categorized based on publication type, with the majority of the studies being journal articles (n=18), followed by conference papers (n=8), and preprints (n=3). The studies used a variety of research designs, including experimental (n=10), qualitative research (n=3), comparative studies (n=4), cross-sectional study (n=2), case study (n=1), observational (n=3), retrospective cohort designs (n=3), mixed methods (n=1), pilot study (n=1), and prospective study (n=1). Table 1 provides more details on the characteristics of the reviewed studies.

**Table 1.** Study characteristics (n=29).

Study	Country	Article category	Study objective	Health care setting	Study design	Evaluation
Montagna et al [6]	Italy	Conference paper	To design and implement a system architecture for a chatbot-based home blood pressure monitoring solution.	Home care setting	Experimental (system design and prototype development)	Human evaluation
Yang et al [16]	China	Preprint	To explore the application of a fine-tuned model-based outpatient treatment support system for treating patients with diabetes and to evaluate its effectiveness and potential value.	Clinical (West China Hospital) home care	Experimental (fine-tuning)	Human evaluation and automated evaluation metrics
Raghu et al [17]	India	Journal article	To evaluate the ability of ChatGPT to predict the diabetic retinopathy risk.	—^a^	Comparative study	Human evaluation
Song et al [18]	The Republic of Korea	Preprint	To investigate the experiences of individuals using LLM^b^ chatbots for mental health support.	Korea Advanced Institute of Science and Technology	Qualitative study	Human evaluation
Liu et al [19]	China	Conference paper	Explore the use of LLMs for cuffless blood pressure measurement using wearable bio signals	Home care settings	Experimental (cuffless blood pressure measurement using LLMs)	Human evaluation
Ogundare et al [20]	Nigeria	Conference paper	To investigate the potential of LLMs in ambulatory devices for sickle cell anemia management.	Home care setting	Case study	Automated evaluation metrics
Cankurtaran et al [21]	Turkey	Journal article	To evaluate the performance of ChatGPT within the context of inflammatory bowel disease.	—	Cross-sectional study	Human evaluation
Wang et al [22]	China	Conference paper	To enhance the diagnosis and treatment of depression.	Clinical and homecare settings	Experimental (pre-training and fine-tuning)	Human evaluation
Abdullahi et al [23]	Germany	Journal article	To explore the potential of three popular Large Language Models in medical education to enhance the diagnosis of rare and complex diseases.	Home care setting	Qualitative study	Human evaluation and automated evaluation metrics
Al Anezi [24]	Saudi Arabia	Journal article	To analyze the use of ChatGPT as a virtual health coach for chronic disease management.	Home care setting	Quasi-experimental design	Human evaluation
Athavale et al [27]	United States	Journal article	To assess whether chatbots could assist with answering patient questions and electronic health record inbox management	Clinical (Division of Vascular Surgery, Stanford University School of Medicine in Palo Alto)	Experimental (chatbot assistance in chronic venous disease management)	Human evaluation
Soto-Chávez et al [28]	Colombia	Journal article	To evaluate the reliability and readability of Spanish chronic disease information presented to ChatGPT	—	Cross-sectional study	Human evaluation
Abbas et al [29]	Pakistan	Journal article	To assess the predictive accuracy of ChatGPT-assisted machine learning models for various chronic diseases.	Clinical (Tertiary hospital)	Observational study	Automated evaluation metrics
Anderson et al [30]	United States	Conference paper	The study aims to discover and rank novel relationships between various aspects of this condition.	—	Experimental	Human evaluation and automated evaluation metrics
Ding et al [31]	Taiwan	Conference paper	To develop and evaluate Large Language Multimodal Models that integrate clinical notes and laboratory test results for predicting the risk of chronic diseases, particularly type 2 diabetes mellitus	Clinical (Eastern Memorial Hospital in Taiwan	Retrospective cohort study	Automated evaluation metrics
Jairoun et al [32]	Malaysia	Journal article	To investigate the benefits and risks associated with the application of ChatGPT in managing diabetes and metabolic illnesses	—	Qualitative study	Human evaluation
Mondal and Naskar [33]	India	Journal article	To evaluate GPT-4’s competency in reviewing diabetic patient management plans compared to expert reviews.	General medical setting	Comparative study	Human evaluation and automated evaluation metrics
Liu et al [34]	N/S^a^	Journal article	To leverage LLMs and multi-prompt engineering for chronic disease management, specifically for detecting mental disorders through user-generated textual content.	Online platforms	Experimental (few-shot learning)	Automated evaluation metrics
Liao et al [35]	Taiwan	Conference paper	To develop an EHR-based chronic disease prediction platform using LLMs for diabetes, heart disease, and hypertension.	Clinical (Far Eastern Memorial Hospital, Taiwan)	Retrospective cohort study	Automated evaluation metrics
Ding et al [36]	Taiwan	Journal article	Predict new-onset type 2 diabetes using large language multimodal models with EHR data	Clinical (Far Eastern Memorial Hospital, Taiwan)	Retrospective cohort study	Automated evaluation metrics
Dao et al [37]	Ireland and Singapore	Conference paper	Design and evaluate an AI chatbot system using GPT-3.5 for proactive diabetes prevention	Community-based setting	Experimental (AI design and evaluation)	Automated evaluation metrics
Khan [38]	United States	Journal article	Assess the efficacy of ChatGPT in facilitating self-management strategies for diabetic patients.	Outpatient diabetes care	Observational study	Human evaluation
Mondal et al [39]	India	Journal article	To evaluate the effectiveness of ChatGPT, an LLM, in providing answers to queries related to lifestyle-related diseases or disorders	Clinical and academic settings	Observational study	Human evaluation
Young et al [40]	United States	Journal article	Assess LLMs’ capacity to deliver age-appropriate explanations of chronic pediatric conditions to enhance patient understanding.	Clinical (Boston Children’s Hospital)	Pilot study	Human evaluation
Li et al [41]	China	Journal article	Develop DeepDR-LLM, an integrated AI system for primary diabetes care and diabetic retinopathy (DR) screening	Low-resource primary care settings	Experimental	Human evaluation and automated evaluation metrics
Ying et al [42]	China	Preprint	To evaluate the feasibility and utility of ChatGPT in diabetes education using retrospective and real-world patient questions.	Outpatient setting	Mixed methods	Human evaluation
Li et al [43]	China	Journal article	To evaluate the performance of LLMs in diabetes-related queries and their potential to assist in diabetes training for primary care physicians	Primary diabetes care, endocrinology, and diabetes management.	Prospective study	Human evaluation and automated evaluation metrics
Hussain and Grundy [44]	Australia	Journal article	Evaluate the responses of ChatGPT models to queries from diabetes patients, assessing their accuracy, biases, and limitations in providing self-management advice.	Home care setting	Comparative study	Human evaluation
Wang et al [45]	China	Journal article	To evaluate the potential of the RISE framework to improve LLMs’ performance in accurately and safely responding to diabetes-related inquiries.	Home care setting	Comparative study	Human evaluation

a Not available.

b LLM: large language model.

As shown in Table 1, most studies were conducted in China (7/29, 24%) and the United States (4/29, 14%), with limited representation of low-resource settings. Experimental designs were predominant (10/29, 35%), and nearly half of the studies (14/29, 48%) focused on diabetes management. The studies primarily focused on adult populations (28/29, 96%), with only 1 study (1/29, 4%) specifically addressing pediatric applications. Human evaluation was the most common evaluation method (16/29, 55%), followed by automated evaluation metrics (10/29, 35%) used in prototype evaluation, with some studies using both approaches (3/29, 10%). Studies were carried out in diverse health care settings, with home care settings (10/29, 35%) and clinical settings (9/29, 31%) being the most common. This diversity in study characteristics reflects the broad application of LLMs across various health care contexts for chronic disease management.

### Methodological Quality Assessment

Using the Mixed Methods Appraisal Tool [26], the studies were categorized as quantitative descriptive studies (19/29, 66%), comprizing observational, cross-sectional, case study, system development, prototype evaluation, and comparative analyses. Quantitative nonrandomized studies (3/29, 10%) included retrospective cohort and quasi-experimental designs, qualitative studies (5/29, 17%), and 2 studies with mixed methods design (2/29, 7%). Of the 29 studies, 18 studies (62.1%) were classified as high quality (meeting ≥4 of 5 criteria), 9 studies (31.1%) as moderate quality (meeting 2‐3 criteria), and 2 studies (6.9%) as low quality (meeting ≤1 criterion). The most common methodological limitations identified across studies included inadequate sampling strategy descriptions, limited participant demographic reporting, use of synthetic clinical data, and lack of external validation. Detailed quality appraisal results for each study are provided in Multimedia Appendix 1. Table 2 provides an overview of the LLM types, users, tasks, and challenges identified across the included studies.

**Table 2.** Large language model tasks and challenges (N=29).

Study	LLM^a^	Users	Disease management tasks	Challenges
Montagna et al [6]	GPT-3	Individuals with hypertension	Patient engagement blood pressure monitoring and management.	The incorrect way patients measure their blood pressure
Yang et al [16]	ChatGLM-6B	Patients diagnosed with diabetes	Treatment recommendations, suggesting appropriate laboratory tests, and medication	Inadequate understanding of complex medical records The small size of the training data
Raghu et al [17]	ChatGPT	Practitioner (ophthalmologist)	Patient education, medical reports: diagnoses and predictions	Incorrect information, privacy and protection of patient data
Song et al [18]	ChatGPT Llama	Individuals who have used LLM chatbots for mental health support	Providing emotional support, engaging in therapeutic conversations, and offering recommendations tailored to individual contexts. Addressing specific stressors or challenges faced by individuals.	Cultural Misalignments: Participants noted that recommendations from LLM chatbots often felt like they were translated from stereotypical American responses. Linguistic Biases: Participants often felt compelled to use English when interacting with LLM chatbots. Therapeutic Misalignment.
Liu et al [19]	Gemma-7B, Mistral-7B, Yi-6B, MedAlpaca-7B, LLaMA2-7B, LLaMA3-8B, Qwen2-7B, PalmyraMed-20B, PMCLLaMA13B, OpenBioLLM-8B	Patients with hypertension	Cardiovascular disease management via cuffless blood pressure measurement.	Dataset imbalances diminish accuracy Privacy concerns in real-world deployment Need for calibration to mitigate individual variability
Ogundare et al [20]	Unspecified	Sickle cell patients and clinicians	Assessing anemia severity in real-time, predicting time to vaso-occlusive episodes, and communicating with emergency personnel.	Creation of a reliable non-invasive tool for angiogenic level assessment, development of a biophysics model, and practical considerations of LLM communication with emergency personnel
Cankurtaran et al [21]	ChatGPT	Inflammatory bowel disease patients and health care professionals.	Tailored responses, educational resources monitoring and follow-up, patient empowerment Decision support	Insufficient responses Limited scope of knowledge (up-to-date information)
Wang et al [22]	LLaMA-7B, ChatGLM-6B Alpaca.	Individuals with depression.	Diagnosis and treatment of depression	Absence of pretraining data sets on depression Hallucination problem Evaluation methodologies emphasize predictive performance and lack Quantification of the impact on patient treatment
Abdullahi et al [23]	Bard ChatGPT 3.5 and GPT-4	Physicians Medical Students Resident Nurses Nurse Practitioners	Clinical Decision Support Medical Education Disease Diagnosis	Inconsistency in Responses, LLMs do not always explicitly indicate their level of uncertainty due to Limited Scope, Sample Size, and knowledge. ChatGPT-3.5 and GPT-4 were limited to health care data available up to 2021 LLMs may generate different responses for the same prompt
Al Anezi [24]	ChatGPT	Outpatients with chronic diseases	Providing information about patient conditions, treatment plans, and medication schedules. Reminders for medication intake, appointments, or lifestyle adjustments. Assisting in behavior change efforts by providing evidence-based strategies, personalized goal-setting techniques, and reminders for healthy habits. Identifying barriers to behavior change and exploring solutions to overcome them. Monitoring blood pressure, blood glucose levels, or weight and providing feedback based on shared data.	Limited physical examination, Lack of human connection and empathy Complexity of individual cases Privacy and security concerns Legal and ethical challenges, language and cultural barriers, technical limitations, diagnostic limitations, and lack of reliability and trust Ineffectiveness in emergencies
Athavale et al [27]	ChatGPT 4.0	Patient	Answered administrative and non-complex medical questions well, and electronic health record inbox management. Answering complex medical questions	Hallucinations Need for extensive supervised training by subject experts No regulatory approval
Soto-Chávez et al [28]	ChatGPT	Patients with chronic diseases using the Spanish language	Evaluating the reliability and readability of ChatGPT-generated patient information on chronic diseases in Spanish.	ChatGPT was trained in English, which affects the accuracy of responses in Spanish Lower reliability on chronic diseases like heart failure and chronic kidney disease
Abbas et al [29]	GPT-3.5	Machine Learning engineers, clinical researchers	Chronic disease prediction	lack of longitudinal data limited generalizability
Anderson et al [30]	GPT (Generative Pre-trained Transformer)	Practitioners	Discover and rank novel relationships between various aspects of chronic lower back pain.	The GPT-based approach took around half an hour to process approximately 500 pairs, making it computationally intensive. Achieving strong agreement among human evaluators
Ding et al [31]	MedAlpaca	Patients with early diabetes Patients with multiclass chronic diseases	Early prediction of diabetes Prediction of multiclass chronic diseases	Lower positive rates when using only laboratory blood values Missing tests for most patients when using only laboratory blood values. Integrating multimodal data from clinical notes and laboratory test results Difficulty in model explainability for early disease prediction
Jairoun et al [32]	ChatGPT	diabetes and metabolic illnesses, endocrinologists and diabetologists	Patient support and education Tailored treatment	Diagnostic mistakes Patient data security and privacy Limitations on generalizability Integration difficulties and workflow errors, and Compliance with laws and regulations Absence of empathy and human contact
Mondal et al [33]	GPT-4	Health care professionals	Reviewing and evaluating diabetes management plans for guideline adherence	GPT-4’s difficulties in handling complex clinical judgments, such as medication adjustments and treatment modifications in varied clinical scenarios
Liu et al [34]	GPT-2 and T5	Patients with mental disorders	Detection of mental disorders (depression, anorexia, pathological gambling, self-harm) through user-generated textual content.	Need for personalized prompts to capture individual user characteristics. Integration of medical knowledge into prompts for accurate detection. Handling noisy and lengthy user-generated content. Few-shot learning with minimal labeled data
Liao et al [35]	BERT BiomedBERT Flan-T5-large-770M GPT-2	Physicians, health care providers	Prediction of chronic diseases (diabetes, heart disease, hypertension) using EHR data.	Difficulty in classifying diseases with lower positive rates (eg, hypertension) Need for interpretability in model predictions Integration of multimodal data (clinical notes and blood test results) for accurate predictions
Ding et al [36]	BERT, Roberta, BiomedBERT, Flan-T5, GPT-2	Researchers, health care professionals	Predict new-onset T2DM, early detection, and risk assessment	Handling multimodal data, missing values, and model interpretability
Dao et al [37]	GPT-3.5	Individuals at risk of diabetes or with prediabetes	Instant Q&A and advice Personalized reminders Data analysis for tailored guidance Health resource aggregation Emotional support	Engagement barriers in prevention programs (eg, transportation, personal responsibilities) Lack of research on AI in diabetes prevention Need for reliable, context-aware AI responses
Khan [38]	ChatGPT	Diabetic patients	Real-time education and support Blood glucose monitoring guidance Medication adherence advice Lifestyle/diet recommendations Emergency detection	Inaccuracies in medical information (eg, insulin storage guidelines, trial data mix-ups) Lack of emotional support/empathy Limited to pre-2021 knowledge Difficulty distinguishing medical terminologies Low adoption among older adults
Mondal et al [39]	ChatGPT-4	Patients and health care professionals	Answering patient questions (causes, symptoms, treatment, diet) Providing information on managing Crohn’s disease (CD) and ulcerative colitis (UC). Addressing professional queries (classification, diagnosis, disease activity, prognostic markers, complications)	Insufficient elaboration on medical agents and surgical indications Inadequate information for patients. ChatGPT provided different answers to the same question across sessions Lower reliability/usefulness scores for patient-directed questions compared to professional-focused ones. Outdated information
Young et al [40]	GPT-4 Gemini 1.0 Ultra	Pediatric patients, health care providers, and caregivers	Generating explanations for chronic conditions	Age-appropriateness discrepancies between models (GPT-4 versus Gemini) Lack of direct feedback from pediatric patients; reliance on clinician evaluations.
li et al [41]	LLaMA	Primary care physicians	Individualized diabetes management recommendations	Underdiagnosis and poor primary diabetes management
Ying et al [42]	GPT-3.5	Physicians, laypersons, and type 2 diabetes patients	Diabetes education and personalized Q&A support	Lower real-world performance, variability by prompt, trust, and safety concerns
Li et al [43]	ChatGPT-3.5 ChatGPT-4.0 Google Bard MedGPT LlaMA2-7B	Researchers Primary care physicians	Answering diabetes-related exam questions and assisting in diabetes training.	Poor performance in both Chinese and English diabetes-related questions LLMs may provide misleading explanations and difficulty with multiple-choice and case analysis questions
Hussain and Grundy [44]	ChatGPT-3.5 ChatGPT-4	Diabetes patients and health care providers	Patient education, treatment recommendations, insulin management, dietary advice	Inaccuracies in medical advice Lack of personalization Failure to recognize regional variations Incorrect assumptions about blood glucose units limitations in addressing complex patient histories
Wang et al [45]	GPT-4 Anthropic Claude 2 Google Bard	Clinicians Diabetes patients	Responding to diabetes-related inquiries and providing accurate and comprehensive information for diabetes self-management.	Lack of specialized medical knowledge in commercially available LLMs Susceptibility to generating inaccurate or misleading information Need for real-time, domain-specific knowledge to improve accuracy and reliability Ensuring responses are safe, accurate, and understandable for patients

a LLM: large language model.

As shown in Table 2, GPT models were the most commonly used (14/29, 48%), followed by LLaMA variants (5/29, 17%), the Bard model (3/29, 10%), and BERT-based models (2/29, 7%). LLMs were primarily used for patient education and information provision (18/29, 62%), with most studies targeting patients (18/29, 62%) rather than health care providers (11/29, 38%). Inaccurate and inconsistencies in responses (18/29, 62 %) were the most frequently reported challenge across studies.

### Objective 1: The Tasks in Chronic Disease Management Performed by LLMs

Our literature synthesis revealed that LLMs have significant potential to improve various chronic disease management tasks. The tasks identified have been broadly categorized into patient-centered tasks and practitioner-centered tasks.

### Patient-Centered Tasks

#### Patient Education and Information Provision

Eighteen studies (n=18) delved into the use of LLMs in providing health information to enhance patient health literacy [16,17,21,23,24,27,28,32,33,37-41]. Key applications included using ChatGPT to provide personalized guidance on diabetes management [16,38,39,42], educational content for diabetic retinopathy and inflammatory bowel disease patients [17,21], generating age-appropriate explanations of chronic pediatric conditions [40], and supporting physician training in diabetes management [43]. In addition, LLMs supported treatment adherence through tools like ChatGPT and GPT-3.5, offering tailored medication reminders, appointment scheduling, and strategies for behavior change [24,37]. For Spanish-speaking populations, ChatGPT was evaluated for reliability and readability of chronic disease information [28], while multiple ChatGPT versions were assessed for regional variations in diabetes education quality [44].

#### Diagnosis and Treatment

Six studies (n=6) examined the role of LLMs in assisting with diagnosis and treatment recommendations. These studies explored the potential of LLMs to suggest appropriate laboratory tests, generating differential diagnoses and medication options tailored to the individual patient’s condition [16,22,32,34]. Notable applications included enhancing depression diagnosis and treatment through fine-tuning of models like LLaMA-7B and ChatGLM-6B [22], supporting the diagnosis of rare and complex diseases [23], and detecting mental disorder patterns through analysis of user-generated content [34]. Furthermore, integrating AI-driven diagnostic and treatment capabilities with diabetes management systems showed particular promise in low-resource primary care settings [41].

#### Self-Management and Disease Monitoring

Eight studies (n=8) addressed using LLMs for self-management and disease monitoring. These studies explored how LLMs provide guidance on managing chronic conditions, promote patient engagement, and support home disease monitoring [6,18,20,21,24,37,38,42]. Key applications included developing chatbot architectures for home blood pressure monitoring [6], creating cuffless blood pressure measurement systems using wearable biosignals [19], and assessing real-time disease severity in sickle cell anemia [20]. LLMs also demonstrated value in detecting emergencies such as hypoglycemic episodes in diabetic patients and guiding appropriate actions [38]. Additional applications encompassed monitoring tools for inflammatory bowel disease management [21], personalized reminders for diabetes prevention [37], and comprehensive health parameter tracking with feedback based on patient-shared data [24]. These implementations highlight the potential of LLMs to enhance patient self-management through continuous monitoring and timely intervention guidance.

#### Emotional Support and Therapeutic Conversations

Four studies (n=4) explored the role of LLMs in providing emotional support and engaging in therapeutic conversations for patients managing chronic diseases. The review identified several key applications, including investigating LLM chatbots for mental health support with tailored recommendations addressing specific stressors [18], evaluating ChatGPT as a virtual health coach identifying barriers to behavior change [24], assessing GPT-3.5’s emotional support capabilities in proactive diabetes prevention [37], and examining ChatGPT’s ability to provide coping strategies for diabetic patients [38].

### Practitioner-Centered Tasks

#### Clinical Decision Support

Eight studies (n=8) investigated the use of LLMs for clinical decision support. The review identified several key applications, including generating personalized medical reports with treatment options and diagnostic procedures for conditions like diabetic retinopathy [17] and inflammatory bowel disease [21], assessing LLMs’ diagnostic accuracy compared with human experts in rare and complex diseases [23], and exploring potential use for electronic health record inbox management [27]. Other applications included using GPT to discover and rank novel relationships between aspects of chronic lower back pain [30], evaluating diabetes management plans using GPT-4 [33], disease classification and prognosis [39], and evaluating LLMs’ competency in answering diabetes-related exam questions for physician training [43].

#### Medical Predictions

Six studies (n=6) explored the predictive capabilities of LLMs in chronic disease management. The review identified several key applications, including predicting diabetic retinopathy risk [17], developing ChatGPT-assisted machine learning models for chronic disease classification [29], and integrating multimodal data from electronic health records and laboratory tests to predict new-onset type 2 diabetes [31,36]. Additional applications included creating an EHR-based prediction platform for diabetes, heart disease, and hypertension [35] and implementing integrated AI systems for diabetes risk assessment in primary care settings [41].

LLMs in chronic disease management are predominantly utilized for patient education and information provision, accounting for (18/29) of reported applications. Self-management and disease monitoring and clinical decision support each account for 28% (8/29) of applications. Diagnosis and treatment tasks, along with medical predictions, both constitute 21% (6/29) of applications, while emotional support and therapeutic conversations account for 14% (4/29). Percentages exceed 100% due to thematic overlaps where individual studies addressed multiple tasks.

LLMs in chronic disease management are predominantly utilized for patient education and information provision, accounting for (18/29) of reported applications. Self-management and disease monitoring and clinical decision support each account for 28% (8/29) of applications. Diagnosis and treatment tasks, along with medical predictions, both constitute 21% (6/29) of applications, while emotional support and therapeutic conversations account for 14% (4/29). Percentages exceed 100% due to thematic overlaps where individual studies addressed multiple tasks.

### Objective 2: Challenges Associated With Using LLMs for Chronic Disease Management

The challenges identified and presented in Table 2 were categorized as follows.

#### Inaccurate and Inconsistencies in Responses

Eighteen studies (n=18) highlighted issues with hallucinations, diagnostic errors, and unreliable outputs [6,16,17,21-23,27-29,32-34,38,39,42-45]. The review identified several key challenges, including hallucinations where models like ChatGPT and LLaMA generate reasonable but factually incorrect information [22,45], diagnostic errors in conditions ranging from depression to inflammatory bowel disease [21,32,44], and inconsistent responses to identical prompts without indicating uncertainty levels [23,39,42]. In addition, models demonstrated limited understanding of complex medical records [16], struggled with regional variations in medical practice [44], provided insufficient elaboration on medical treatments [39], and showed difficulty distinguishing medical terminologies [38]. Further challenges included lower reliability in the Spanish language for specific chronic conditions like heart failure and chronic kidney disease [28] as well as limited generalizability due to restricted training populations [29,32]. These inaccuracies stem from erroneous input data, such as incomplete or incorrect test results [6,21,22,27,31].

#### Limited Datasets and Knowledge

Six studies (n=6) identified challenges related to limited datasets and knowledge cutoffs in LLM applications for chronic disease management. The review highlighted several key limitations, including scarcity of disease-specific datasets [16,22], dataset imbalances affecting predictions for conditions like hypertension [19], and knowledge limitations that restrict LLM awareness of the current medical guidelines [21,23,39].

#### Computational and Technical Challenges

Six studies (n=6) highlight significant computational and technical challenges in deploying LLMs for chronic disease management. The review identified several key limitations, including resource-intensive processing that results in prolonged training time in resource-constrained environments [30]. In addition, technical challenges include integrating multimodal data from clinical notes and laboratory results [31,36], ensuring model explainability for early disease prediction [35], and handling noisy user-generated content in mental health applications [31,34]. Further challenges involve difficulties in integrating LLMs into clinical workflows [32] and managing complex clinical judgments, such as medication adjustments and treatment modifications [33].

#### Usability and Accessibility Concerns

Nine studies (n=9) identified usability and accessibility concerns surrounding LLMs in chronic disease management tasks. Notably, the restriction to textual inputs limits use for tasks involving multimodal diagnostic tasks [28,40] language and cultural misalignments [18,28], while age-inappropriate outputs pose challenges for pediatric care [40]. In addition, poor interpretability of model predictions for early disease prediction and risk assessment [31,35,36]. Additional challenges included a lack of empathy and ineffectiveness in emergencies [24,29], digital literacy gaps restricting adoption among older adults [29,38]. Furthermore, these studies also noted how insufficient transparency in model decision-making processes hindered trust and clinical acceptance [35,36].

#### LLM Evaluation

Five studies (n=5) noted challenges involving LLM evaluation. Notable challenges identified included that automated evaluation metrics primarily focus on predictive performance and fail to assess the impact on patient treatment outcomes [22], difficulties in achieving consensus among human evaluators when assessing LLM outputs [30], discrepancies between model performance in test environments versus real-world applications [42], difficulties in consistently evaluating language models across different diabetes-related tasks [43], and significant variations in age-appropriateness scoring between different LLM platforms [40].

#### Legal, Ethical, Privacy, and Regulatory Concerns

Ten studies (n=10) identified legal, ethical, privacy, and regulatory challenges of using LLMs in chronic disease management. The review highlighted several critical concerns, including privacy and data security vulnerabilities [17,19,24,27,28,32], absence of regulatory approval and standardized guidelines [27], and compliance issues with health care laws across different jurisdictions [27,32]. In addition, language and cultural barriers posed additional challenges, particularly for non-English speakers [18,28], while bias and equity issues stemming from limited training data diversity raised concerns about health care disparities [24,32,42]. Studies also noted ethical challenges around accountability for errors [24], lack of transparency in decision-making [22,27], and limitations in addressing complex ethical dilemmas in clinical care [44,45].

The most prevalent challenge identified was inaccurate and inconsistent responses, reported in 62% (18/29) of studies. Legal, ethical, privacy, and regulatory concerns followed, appearing in 35% (10/29) of studies. Usability and accessibility issues were noted in 31% (9/29) of studies. Computational and technical limitations, as well as dataset and knowledge constraints, were each reported in 21% (6/29) of studies. Additionally, 17% (5/29) of studies highlighted limitations in evaluation methodologies.

## Discussion

### Principal Findings

This scoping review presents 3 significant findings from the 29 included studies (n=29): (1) LLMs are mostly used for both patient-centered (18/29, 62%) and practitioner-centered (11/29, 38%) tasks, with patient education and information provision emerging as the major application (18/29, 62%); (2) despite promising applications, significant challenges still exist, particularly regarding LLM response accuracy (18/29, 62% of studies), ethical concerns (10/29, 35%), and usability issues (9/29, 31%); and (3) methodological quality varies considerably across studies, with journal articles demonstrating higher quality (13/18, 72%) compared with conference papers (3/8, 38%) and preprints (1/3, 33%). These findings highlight both the considerable promise and significant limitations of current LLM applications in chronic disease management, which are examined in detail below.

### Chronic Disease Management Tasks

Chronic disease management is an approach to managing chronic illnesses involving screenings, regular check-ups, monitoring, coordination of treatment, medication adherence, lifestyle modifications, and patient education [1]. The findings from this scoping review reveal an increasing interest in leveraging LLMs like ChatGPT to support both patient-centered and practitioner-centered tasks [6,16,22-24,30,31].

### Patient-Centered Tasks

The majority of the studies (18/29, 62%) focused on patient-centered tasks, reflecting the emphasis on patient active engagement in chronic disease management [3,46]. Patients’ active engagement enables them to monitor their symptoms, disease progress, weight, and adverse drug effects, and adhere to medication and visits [3,46]. This review found that LLMs support various patient-centered applications, including patient education and information provision, disease monitoring and self-management, emotional support and therapeutic conversations, and diagnosis and treatment assistance.

Patient education and information provision emerged as the most prominent application (18/29, 62%), with LLMs providing health information about conditions, treatment plans, and medication schedules [16,17,21,23,24,27,28,32,33,37-41]. With the right health information, individuals with chronic diseases can easily self-manage their conditions. Diagnosis and treatment applications accounted for (6/29, 21%). Studies experimented using LLMs to suggest laboratory tests, for diagnosis, and for medication generation tailored to individual patient conditions [16,22,23,32,34,41]. However, using LLMs for diagnostic and treatment has been criticized due to concerns about hallucinations and misinterpretations of clinical guidelines [22,23], highlighting the need for continued research to ensure patient safety. Therefore, LLM usage should not replace health practitioners but instead serve as complementary tools.

Self-management and disease monitoring applications (8/29, 28%) demonstrated how LLMs can facilitate home-based monitoring of various physiological parameters [6,19-21,24,37,38,42]. Recent studies have highlighted that patient engagement with health monitoring technologies is crucial for improving health outcomes in chronic disease management [47]. Studies have also shown that wearable technologies integrated with LLMs provide real-time patient-centered health data that can better inform self-management decision-making [48]. However, ensuring consistent long-term engagement is still a challenge.

Emotional support and therapeutic conversations (4/29, 14%) represented an emerging application area [18,24,37,38]. Studies showed that LLMs can provide psychological support through tailored recommendations addressing specific stressors [18], identifying barriers to behavior change [24], and offering coping strategies for patients with diabetes [38]. Emotional support is increasingly recognized as essential in chronic disease management [49], which helps patients overcome psychological barriers to treatment adherence and lifestyle modifications.

### Practitioner-Centered Tasks

Practitioner-centered tasks (11/29, 38%) mainly revolved around clinical decision support and medical predictions. Clinical decision support applications (8/29, 28%) provided health care practitioners with actionable information to enhance decision-making. Studies demonstrated that LLMs can generate personalized medical reports, generate treatment recommendations, and support diagnostic processes that assist health care specialists in making informed decisions [17,21]. However, while LLMs enhance diagnostic efficiency, concerns regarding inconsistent outputs pose barriers to clinical adoption. Medical prediction applications account for (7/29, 24%) of LLM use in chronic disease management, showing strong potential for early disease detection and risk stratification. By integrating structured and unstructured clinical data, such as lab results, clinical notes, and imaging, LLMs enable more comprehensive and accurate predictive models compared with traditional methods [17,29,31,34-36].

Notably, real-time risk assessment tools, like the ambulatory device developed for sickle cell anemia management [20], demonstrate how LLMs can predict complications before symptoms appear. However, challenges relating to medical accuracy still limit their seamless integration into clinical workflows [6,16,17,21-23,27]. A significant emerging trend is the development of retrieval-augmented generation (RAG) frameworks that enhance prediction accuracy by dynamically incorporating up-to-date medical knowledge [22,35,50-53]. Future advancements should focus on seamless integration of LLMs with existing medical systems, wearable health technologies, and mobile health applications to improve interoperability and trustworthiness.

### Methodological Quality and Strength of Evidence

The methodological quality assessment revealed notable trends that should be considered while interpreting the results of this scoping review. High-quality studies (18/29, 62%) were not uniformly distributed across LLM application tasks, studies examining medical prediction applications [29,31,35,36] and patient education and information provision [28,32,38,39] had significantly stronger methodological rigor. Studies investigating emotional support showed mixed quality, with some high-quality qualitative research [18,24], a quantitative descriptive study [37] alongside a moderate-quality mixed study [38]. Notably, self-management and disease monitoring studies showed significant methodological heterogeneity, with quality ratings ranging from low to high.

Journal articles demonstrated a substantially higher proportion of high-quality studies (13/18, 72%) compared with conference papers (3/8, 38%) and preprints (1/3, 33%), suggesting that peer-review processes enhance methodological quality. The identified common limitations, including inadequate sampling strategies, limited participant demographic reporting, and insufficient methodological transparency, were more prevalent in lower-tier publication sources, including conference papers and preprints. Quantitative descriptive studies, especially those focused on system design and prototype testing (10/29, 34.5%), showed mixed quality ratings ranging from low to high, with a common limitation being the use of synthetic data, lack of clinical validation, as they commonly prioritized technical utility. These methodological patterns significantly influence the reliability of the findings.

### Challenges and Corresponding Recommendations

This section discusses the key challenges identified in the review and presents corresponding recommendations to mitigate these issues. Each challenge is followed by practical solutions to enhance the applicability of LLMs in chronic disease management.

### Inaccurate Data and Inconsistencies in Responses

Inaccurate and inconsistent responses emerged as a significant challenge (18/29, 62%), primarily due to poor data quality, inherent biases in training datasets, and the limited scope of knowledge constrained by the model’s training cutoff [6,22,23,31]. Given that LLM performance is intrinsically linked to data quality, flawed datasets inevitably propagate errors in outputs, a manifestation of the “garbage in, garbage out” principle [54-56]. The issue of biases in AI models has gathered significant attention in recent literature [54-56], prompting possible migration strategies [57-59]. These limitations carry critical implications for health care, as erroneous LLM outputs may lead to incorrect clinical decisions, posing significant risks to patient safety [8,12]. Hence, researchers are exploring technical solutions such as advancing domain-specific fine-tuning techniques [60,61], leveraging retrieval-augmented generation (RAG) frameworks [22,50-52], and refining outputs through reinforcement learning (RL) and prompt engineering techniques [62-64]. In addition, implementing expert validation protocols has emerged as a crucial safeguard to ensure adherence to evidence-based practice.

### Limited Datasets

The scarcity of high-quality datasets for chronic diseases accounted for (6/29, 21%). Studies highlighted limitations and narrow coverage of publicly accessible clinical training datasets due to data privacy and institutional restrictions [16,19,21-23,39]. Given that experimental studies often require substantial model training datasets, the absence of adequate data poses a significant challenge to the effectiveness and success of these studies. Therefore, to address this gap, synthetic datasets and data augmentation techniques have been explored by studies [15,65]. However, these methods risk amplifying pre-existing biases in source data [54-56]. Therefore, dataset validation is essential to ensure quality, collaborative partnerships with health care institutions to access real-world clinical datasets and knowledge distillation techniques, where smaller models can be trained on outputs from larger, clinically validated models, reducing dependency on raw data volume, can be explored [66].

### Computational Resources and Technical Challenges

The computational demands of training LLMs for health care applications remain a significant challenge (6/29, 21%). Consequently, low computing resources approaches, such as quantization, parameter-efficient fine-tuning (PEFT) techniques like Low-Rank Adaptation (LORA), Quantized LoRA (QLORA), Weight-Decomposed Low-Rank Adaptation, and REFT [67-73], are evolving and are popularly used in fine-tuning LLMs in low-resource computing environments. In addition, adapter-based tuning methods provide lightweight alternatives by injecting trainable adapter layers into frozen pretrained models, enabling task-specific fine-tuning without updating the entire model parameters [74,75]. Building on these advances, the development of lightweight LLMs optimized for mobile devices presents a promising direction for extending AI-based chronic disease management to resource-constrained settings [76].

### Usability and Accessibility Concerns

Usability and accessibility concerns accounted for (9/29, 31%), including issues with text-only interfaces for some LLMs, cultural misalignments, and outputs ill-suited for pediatric or elderly populations [18,28,40]. While studies highlighted text-only interfaces as a critical limitation of LLMs in health care [28,40], recent advances in multimodal architectures have addressed this gap. These models now integrate and interpret diverse data modalities, including medical images, audio, and structured documents, while generating composite textual and visual outputs [31,77-79]. In addition, having dynamic and simplified interfaces to accommodate low-digital-literacy users, pediatric users, and for different cultural and language settings could further improve LLM usability and adaptation.

### Legal, Ethical Issues, and Regulatory Issues

Legal, ethical, and regulatory concerns (10/29, 35%) remain a key issue in LLM adoption in health care. Studies identified data privacy, biases, misinformation, responsibility, and accountability for LLM-generated content as key concerns. Although several AI frameworks have been proposed, the absence of standardized guidelines and regulatory approvals creates significant gaps [27,80-82]. This regulatory vacuum risks inconsistent model development and validation practices, unaddressed ethical dilemmas regarding accountability for AI-generated recommendations, and potential mismatches between rapidly evolving LLM capabilities and static health care regulations [27,83]. Addressing these ethical concerns requires standard guidelines and rules to ensure responsible use in health care settings[84]. Therefore, future efforts must prioritize the development of a comprehensive regulatory framework.

### LLM Evaluation

Evaluation gaps accounted for (5/29, 17%), reflecting critical shortcomings in current methodologies for assessing LLM performance in clinical contexts. The existing automated evaluation metrics mainly focus on predictive performance using metrics such as accuracy and *F*_1_-scores that lack medical and treatment knowledge [22,85]. These metrics may produce misleading conclusions if not appropriately selected [85]. Furthermore, human evaluation, although valuable, introduces subjectivity and interrater variability, yet the absence of a standardized LLM evaluation framework makes attaining consensus among human raters challenging [30,85]. A promising direction involves a hybrid evaluation approach that integrates human expert reviews with automated metrics. Future efforts should prioritize the development of standardized LLM evaluation frameworks tailored to health care settings. Table 3 summarizes the challenges associated with applying LLMs in chronic disease management with proposed recommendations.

**Table 3.** Key challenges and recommendations.

Challenge	Key observations	Recommendation
Inaccurate and inconsistent responses	Hallucinations, inconsistent responses, outdated knowledge	Adopting RAG frameworks, fine-tuning, and prompt engineering. RL, expert validation of LLM-generated recommendations
Limited datasets	Scarcity of datasets, missing data, and dataset imbalances.	Use synthetic data, data augmentation, partnerships with health care institutions, knowledge distillation
Computational demands	High computational demands	Adopt PEFT (LORA and QLORA), quantization use lightweight models for mobile devices
Ethical and privacy concerns	Privacy concerns, language and cultural barriers, and lack of regulatory oversight	Develop a regulatory framework
Usability issues	Restriction to textual inputs, lack of empathy, ineffectiveness in emergencies Age-appropriate interaction	Use multimodal LLMs. Dynamic interfaces to accommodate low-digital-literacy users, age-appropriate interaction modes customize to different cultural settings Integrate with wearable devices and mobile health apps
Evaluation challenges	Predictive performance metrics, subjectivity, and interrater variability	Develop a standardized LLM evaluation framework for health care.

### Limitations of the Study

Although quality assessment was conducted using MMAT, studies were not excluded based on their methodological rigor. As a result, including moderate and low-quality studies may have influenced the reliability and consistency of the reviewer’s findings. The varied methodological designs across studies may have affected the interpretation of results and conclusions drawn. Furthermore, the review was limited to only English-language publications, which may have introduced language bias and limited the generalizability of our findings, particularly in contexts where LLMs are being adapted to local languages or integrated into culturally specific health care practices. The exclusion of databases such as Embase and Web of Science may have limited the comprehensiveness of the search. Future research can consider broader database coverage and non-English sources to enhance diversity and scope.

### Implications for Practice and Future Research

This scoping review reveals several critical implications for the integration of LLMs in chronic disease management. First, it is essential to address the accuracy issues identified in 18/29, 62% of studies. This calls for both technical solutions (domain-specific fine-tuning, reinforcement learning (RL), and retrieval-augmented generation (RAG) frameworks [61,64]) and nontechnical solutions (expert validation and collaborative partnerships with health care institutions to access and use real-world clinical data). Second, enhancing accessibility across diverse patient populations requires developing culturally adapted LLM interfaces, implementing age-appropriate interaction modes [40], and integrating with low-resource platforms such as SMS-based systems and lightweight mobile apps for various populations [76]. Third, robust governance frameworks must be established to address the ethical, legal, and privacy concerns noted in 10/29, 35% of studies to ensure regulatory compliance.

Finally, future research should focus on multimodal LLMs that can synthesize diverse data inputs from wearable devices and mobile health applications for holistic patient monitoring [19,31,35] and develop resource-efficient deployment techniques. The predominance of diabetes-focused studies (14/29, 48%) highlights a potential research gap in addressing other prevalent chronic conditions. Similarly, there is a research gap in age-inclusive LLM design, given the overwhelming focus on adult populations (28/29, 96%) and the lack of pediatric studies. Addressing these gaps would enhance the clinical relevance and equitable application of LLMs across the full spectrum of chronic disease management.

### Conclusion

This scoping review highlights the growing potential of LLMs in supporting chronic disease management through patient education, diagnosis and treatment, emotional support, self-management support, decision support, and prediction tasks. While LLMs offer promising capabilities, their effective integration into health care still requires addressing key challenges related to accuracy, accessibility, usability, and ethical and privacy concerns. Future research should focus on integrating LLMs with mobile and wearable technologies, creating culturally and age-appropriate interfaces, and exploring integration with low-resource platforms. Addressing these research gaps will ensure equitable and safe use of LLMs across diverse health care settings.

## Supplementary material

10.2196/66905 Multimedia Appendix 1 Detailed methodological quality appraisal results for each included study using the Mixed Methods Appraisal tool (MMAT).

10.2196/66905 Checklist 1 PRISMA-ScR checklist.

## Abbreviations

LLM: large language model
PRISMA-ScR: Preferred Reporting Items for Systematic Reviews and Meta-Analyses Extension for Scoping Reviews
RAG: retrieval augmented generation
RL: reinforcement learning
