# Supplementary material for: Using Large Language Models for Chronic Disease Management Tasks: Scoping Review
Source: JMIR Med Inform. 2025 Sep 29;13:e66905. doi: 10.2196/66905 (PMC12479051; doi:10.2196/66905)
Supplement: Multimedia Appendix 1 [file medinform-v13-e66905-s001.docx]

**Table S1.**

| **Study** | **Design** | **MMAT Criteria Met** (✓/✗)  (4.1-4.5) | **Quality Rating** | **Key Limitations** |
| --- | --- | --- | --- | --- |
| Montagna et al. (2023) [6] | Quantitative Descriptive | ✗✗✓✗✗ **(1/5)** | Low | No participant sampling or characterization; no statistical analysis; limited user engagement data; purely descriptive prototype use case; no reported outcomes or metrics. |
| Wang et al. (2023) [25] | Quantitative Descriptive | ✗✗✓✗✓ **(2/5)** | Moderate | No clinical trial validation Unclear demographic diversity Black-box LLM use (Limited interpretability of diagnostic logic). No mention of IRB/consent for patient data |
| Al Anezi. (2023) [27] | Qualitative study | ✓✓✓✓✓ **(5/5)** | High | Limited to EHR data; potential risks with complex cases; cannot fully replace clinical judgment. |
| Yang et al. (2023) [19] | Quantitative Descriptive | ✗✗✓✗✓ **(2/5)** | Moderate | Lack of detailed sampling strategy and participant demographics; limited information on response rates |
| Athavale et al. (2023) [29] | Quantitative Descriptive | ✓✗✓✗✓ **(3/5)** | Moderate | Limited sample size, lack of detailed participant demographics |
| Anderson et al. (2024) [32] | Quantitative Descriptive | ✓✓✗✗✓ **(2/5)** | Moderate | Lack of a detailed sampling strategy and participant demographics  Limited information on response rates |
| Abdullahi et al. (2024) [26] | Qualitative | ✓✓✓✓✓ **(5/5)** | High | Uses simulated public cases |
| Young et al. (2024) [42] | Quantitative Descriptive | ✓✓✓✓✗ **(4/5)** | High | Small pediatric sample (n=20) Short-term outcomes only |
| Liu et al. (2024) [22] | Quantitative Descriptive | ✓✓✓✓✗ **(4/5)** | High | No external validation |
| Hussain & Grundy (2025) [46] | Quantitative Descriptive | ✓✗✓✗✓ **(3/5)** | Moderate | Small, convenience sample; no diversity analysis; limited longitudinal impact data. |
| J. E. Ding et al. (2024)[33] | Quantitative non-randomized | ✓✓✓✓✗ **(4/5)** | High | Lack of external validation |
| Cankurtaran et al. (2023) [24] | Quantitative  Descriptive | ✓✓✓✗✓ **(4/5)** | High | Small sample (n=35) |
| Song et al, (2024) [21] | Qualitative | ✓✓✓✓✓ **(5/5)** | High | Self-reported data; small sample size; lacks clinical outcome evaluation. |
| Ogundare et al. (2023) [23] | Qualitative | ✓✗✗✗✗ **(1/5)** | Low | Theoretical framework without empirical validation |
| Jairoun et al. (2024) [34] | Qualitative | ✓✓✓✓✓ **(5/5)** | High | None |
| Soto-Chávez et al. (2023) [30] | Quantitative Descriptive | ✓✓✓✗✓ **(4/5)** | High | Used simulated cases |
| Raghu et al. 2023 [20] | Quantitative Descriptive | ✓✗✓✗✓ **(3/5)** | Moderate | Limited sample representativeness |
| Mondal & Naskar (2024)[35] | Quantitative Descriptive | ✓✓✓✗✓ **(4/5)** | High | Used simulated cases rather than real-world clinical data |
| Dao et al. (2024) [39] | Quantitative Descriptive | ✓✓✓✗✓ **(4/5)** | High | None |
| Wang D et al (2024) [47] | Quantitative Descriptive | ✓✓✓✗✗ **(3/5)** | Moderate | Used only synthetic data |
| Abbas et al. (2024) [31] | Quantitative Descriptive | ✓✓✓✓✓ **(5/5)** | High | None |
| Ying et al. (2023) [44] | Mixed methods | ✓✗✓✗✓ **(3/5)** | Moderate | Lower ratings in real-world evaluations; performance depends on prompt quality; limited interactions. Limited transparency in prompt engineering |
| Liu et al. (2023) [36] | Quantitative Descriptive | ✓✓✓✓✗  **4/5** | High | Lab-controlled BP measurements only |
| Liao et al. (2024) [37] | Quantitative  non-Randomized | ✓✓✓✗✓  **4/5** | High | Early prototype |
| J. E. Ding et al. (2024) [38], | Quantitative  Non-Randomized | ✓✓✓✓✓  **5/5** | High | None |
| li et al. (2024) [43] | Quantitative Descriptive | ✓✓✓✓✓  **5/5** | High | None |
| H. Li et al. (2025) [45] | Quantitative Descriptive | ✓✓✓✓✓  **5/5** | High | None |
| Khan (2023) [40] | Mixed methods | ✓✗✓✗✓  **3/5** | Moderate | Limited sample diversity |
| Mondal et al [41], 2023 | Quantitative Descriptive | ✓✓✓✓✓  **5/5** | High | None |

✓ = criterion met, ✗ = not met

**References**

6. Montagna S, Ferretti S, Klopfenstein LC, Florio A, Pengo MF. Data decentralisation of LLM-based chatbot systems in chronic disease self-management. Presented at: ACM International Conference Proceeding Series. 205-212; Sep 6, 2023.[doi: 10.1145/3582515.3609536]

19. Raghu K, S T, S Devishamani C, M S, Rajalakshmi R, Raman R. The utility of ChatGPT in diabetic retinopathy risk assessment: a comparative study with clinical diagnosis [response to letter]. Clin Ophthalmol. 2024;18:313-314. [doi: 10.2147/OPTH.S461186] [Medline: 38317795]

20. Song I, Pendse SR, Kumar N. The typing cure: experiences with large language model chatbots for mental health support. Preprint posted online on 2024.

21. Liu Z, Chen C, Cao J, et al. Large language models for cuffless blood pressure measurement from wearable biosignals. Presented at: Proceedings of the 15th ACM International Conference on Bioinformatics, Computational Biology and Health Informatics. 1-11; Nov 22, 2024.[doi: 10.1145/3698587.3701447]

22. Ogundare O, Sofolahan S. Large language models in ambulatory devices for home health diagnostics: a case study of sickle cell anemia management. In: Advances in Intelligent Networking and Collaborative Systems. Vol 182. Lecture Notes on Data Engineering and Communications Technologies; 2023:447-453. [doi: 10.1007/978-3-031-40971-4_42]

23. Cankurtaran RE, Polat YH, Aydemir NG, Umay E, Yurekli OT. Reliability and usefulness of ChatGPT for inflammatory bowel diseases: an analysis for patients and healthcare professionals. Cureus. Oct 2023;15(10):e46736. [doi: 10.7759/cureus.46736] [Medline: 38022227]

24. Wang X, Liu K, Wang C. Knowledge-enhanced pre-training large language model for depression diagnosis and treatment. Presented at: 2023 IEEE 9th International Conference on Cloud Computing and Intelligent Systems (CCIS). 532-536; 2023.[doi: 10.1109/CCIS59572.2023.10263217]

25. Abdullahi T, Singh R, Eickhoff C. Learning to make rare and complex diagnoses with generative AI assistance: qualitative study of popular large language models. JMIR Med Educ. Feb 13, 2024;10:e51391. [doi: 10.2196/51391] [Medline: 38349725]

26. Al-Anezi FM. Learning Health Systems - 2024 - Al‐Anezi - Exploring the use of ChatGPT as a virtual health coach for chronic disease.pdf,” learning health systems. 2023. [doi: 10.1002/lrh2.10406]

27. Tricco AC, Lillie E, Zarin W, et al. PRISMA extension for scoping reviews (PRISMA-ScR): checklist and explanation. Ann Intern Med. Oct 2, 2018;169(7):467-473. [doi: 10.7326/M18-0850] [Medline: 30178033]

29. Athavale A, Baier J, Ross E, Fukaya E. The potential of chatbots in chronic venous disease patient management. JVS Vasc Insights. 2023;1:100019. [doi: 10.1016/j.jvsvi.2023.100019] [Medline: 37701430]

30. Soto-Chávez MJ, Bustos MM, Fernández-Ávila DG, Muñoz OM. Evaluation of information provided to patients by ChatGPT about chronic diseases in Spanish language. Digit Health. 2024;10:20552076231224603. [doi: 10.1177/20552076231224603] [Medline: 38188865]

31. Abbas S, Iftikhar M, Shah MM, Khan SJ. ChatGPT-assisted machine learning for chronic disease classification and prediction: a developmental and validation study. Cureus. Dec 2024;16(12):e75851. [doi: 10.7759/cureus.75851] [Medline: 39822450]

32. Anderson P, et al. Bridging domains in chronic lower back pain: large language models and ontology-driven strategies for knowledge graph construction. 2024.

33. Ding JE, Thao PHM, et al. Large language multimodal models for 5-year chronic disease cohort prediction using EHR data. 2024:1-10.

34. Jairoun AA, Al-Hemyari SS, Shahwan M, Al-Qirim T, Shahwan M. Benefit-risk assessment of ChatGPT applications in the field of diabetes and metabolic illnesses: a qualitative study. Clin Med Insights Endocrinol Diabetes. 2024;17:11795514241235514. [doi: 10.1177/11795514241235514] [Medline: 38495947]

35. Mondal A, Naskar A. Artificial intelligence in diabetes care: evaluating GPT-4’s competency in reviewing diabetic patient management plan in comparison to expert review. Endocrinology (including Diabetes Mellitus and Metabolic Disease). 2024. [doi: 10.1101/2024.04.12.24305732]

36. Liu H, Zhang W, Xie J, et al. Few-shot learning for chronic disease management: leveraging large language models and multi-prompt engineering with medical knowledge injection. Presented at: Hawaii International Conference on System Sciences. [doi: 10.24251/HICSS.2025.084] [Medline: 38681743]

37. Liao C, Kuo W, Hu I, et al. EHR-based mobile and web platform for chronic disease risk prediction using large language multimodal models. Association for Computing Machinery. 5244-5248; 2024.[doi: 10.1145/3627673.3679227.]

38. Ding JE, Thao PNM, Peng WC, et al. Large language multimodal models for new-onset type 2 diabetes prediction using five-year cohort electronic health records. Sci Rep. Sep 6, 2024;14(1):20774. [doi: 10.1038/s41598-024-71020-2] [Medline: 39237580]

39. Dao D, Teo JYC, Wang W, Nguyen HD. LLM-powered multimodal AI conversations for diabetes prevention. Presented at: ICMR ’24. 1-6; Jun 10, 2024.[doi: 10.1145/3643479.3662049]

40. Khan M. Assessing the efficacy of ChatGPT in facilitating self-management strategies among diabetic patients section a-research paper 10490 Eur. [doi: 10.48047/ecb/2023.12.10.744]

42. Young CC, Enichen E, Rao A, et al. Pilot study of large language models as an age-appropriate explanatory tool for chronic pediatric conditions. Pediatrics. [doi: 10.1101/2024.08.06.24311544]

43. Li J, Guan Z, Wang J, et al. Integrated image-based deep learning and language models for primary diabetes care. Nat Med. Oct 2024;30(10):2886-2896. [doi: 10.1038/s41591-024-03139-8] [Medline: 39030266]

44. Ying Y, Wang Y, Yuan S, et al. Exploration of chatgpt application in diabetes education based on multi-dataset. medRxiv. Preprint posted online on 2023. [doi: 10.1101/2023.09.27.23296144]

45. Li H, Jiang Z, Guan Z, et al. Large language models for diabetes training: a prospective study. Sci Bull Sci Found Philipp. Mar 2025;70(6):934-942. [doi: 10.1016/j.scib.2025.01.034]

46. Hussain W, Grundy J. Advice for diabetes self-management by chatgpt models: challenges and recommendations. Preprint posted online on Jan 2025.

47. Wang D, Liang J, Ye J, et al. Enhancement of the performance of large language models in diabetes education through retrieval-augmented generation: comparative study. J Med Internet Res. Nov 8, 2024;26:e58041. [doi: 10.2196/58041] [Medline: 39046096]
